# Supplementary material for: Analysing the effect of full-length and C-terminally truncated progranulin on proliferation, colony formation, and migration in HepG2 and U87 cells
Source: Sci Rep. 2025 Nov 28;15:42698. doi: 10.1038/s41598-025-26703-9 (PMC12663392; doi:10.1038/s41598-025-26703-9)
Supplement: Supplementary file 5 — Supplementary Material 5 [file 41598_2025_26703_MOESM5_ESM.pdf]

Supplementary Figures S1-S3 for Hofer et al., *Analysing the effect of full-length and C-terminally truncated progranulin on proliferation, colony formation, and migration in HepG2 and U87 cells*

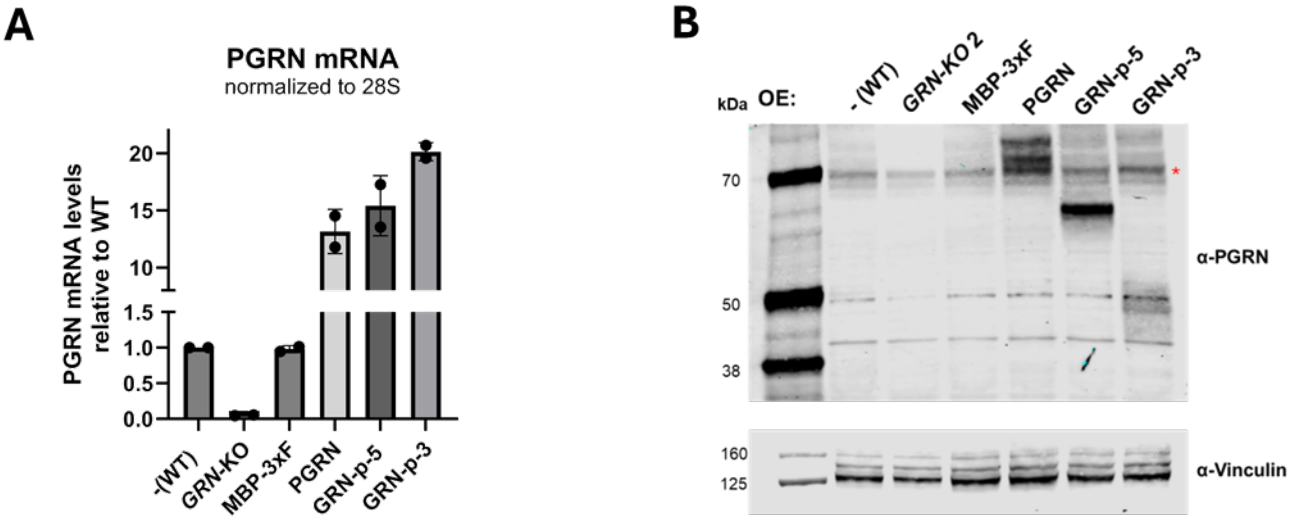

Supplementary Figure 1: Analysis of PGRN-overexpression levels. *(A) RT-qPCR analysis of the progranulin mRNA levels (n=2). (B) Progranulin western blot using the monoclonal sc-377036 anti-PGRN antibody from Santa-Cruz Biotech. which supposedly only binds granulin-2. Asterix indicates an unspecific band just below the full-length PGRN band.*

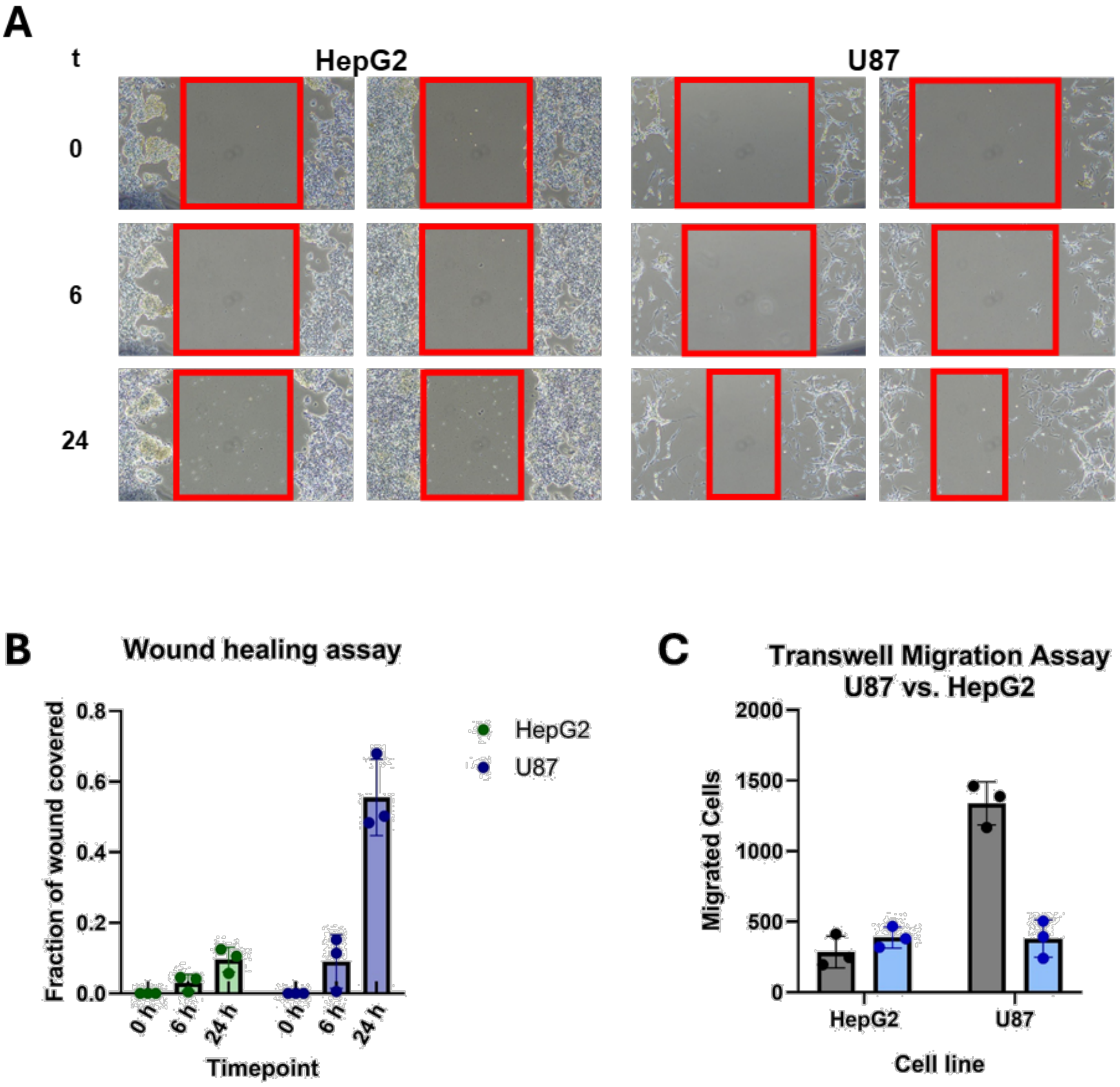

Supplementary Figure S2: Migration of HepG2 and U87 cells in comparison. *(A) Representative scratch (Wound healing) assay of HepG2 and U87 cells. Cells were grown to high confluency, and then a thin line of cells was scratched off the plate with a pipette tip. The population of this cell-free area with cells was monitored for 24 hours in serum-free medium. (B) Quantification of wound healing assay to compare migration ability of HepG2 and U87 cells (n=3). (C) Transwell migration assay assessing the migration of HepG2 and U87 cells towards medium containing FCS (+FCS) and random migration towards serum-free medium (-FCS) after 24 hours (n=3). B and C were created using GraphPad Prism.*

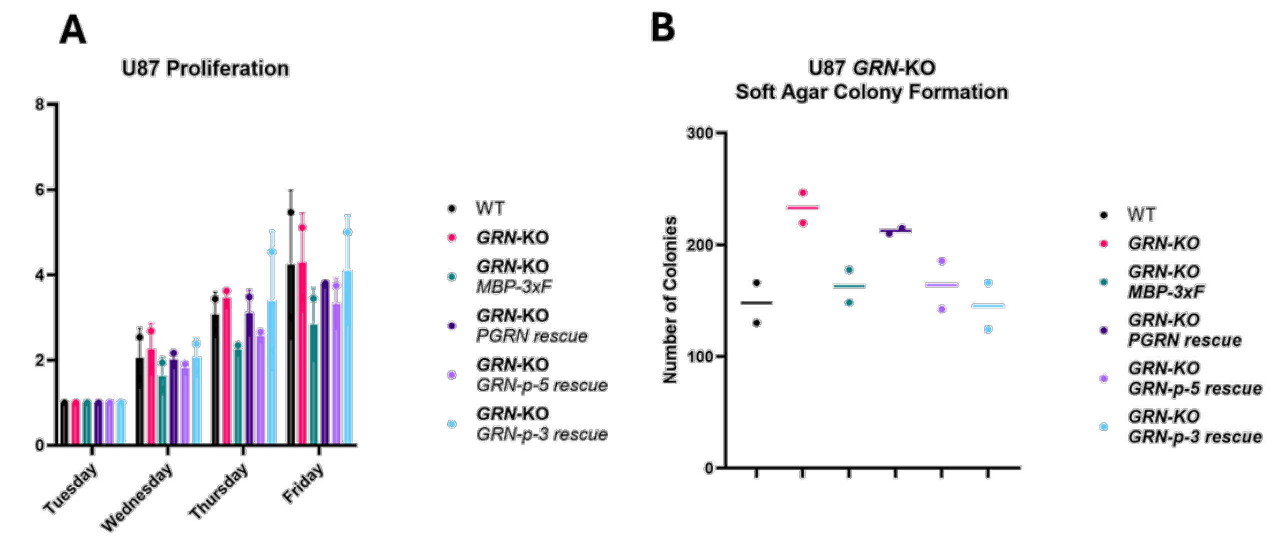

Supplementary Figure S3: GRN-KO and PGRN-rescue in U87 cells. *(A) Proliferation of U87 GRN-KO and rescue cell lines and (B) Soft-agar colony-formation assay (n=2). Panels were created with GraphPad Prism.*
