# Supplementary material for: Analysing the effect of full-length and C-terminally truncated progranulin on proliferation, colony formation, and migration in HepG2 and U87 cells
Source: Sci Rep. 2025 Nov 28;15:42698. doi: 10.1038/s41598-025-26703-9 (PMC12663392; doi:10.1038/s41598-025-26703-9)

Raw Image Figure 2A: α-PGRN (AF2420), α-Flag

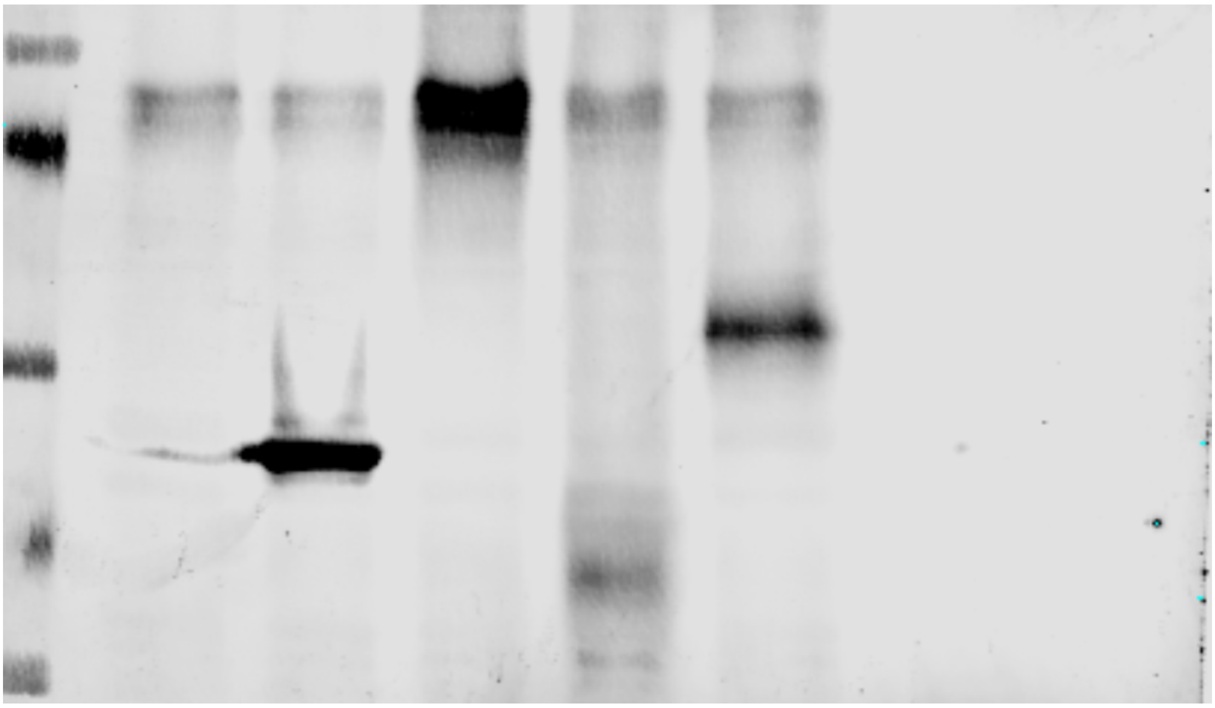

Raw Image Figure 2A: α-Vinculin

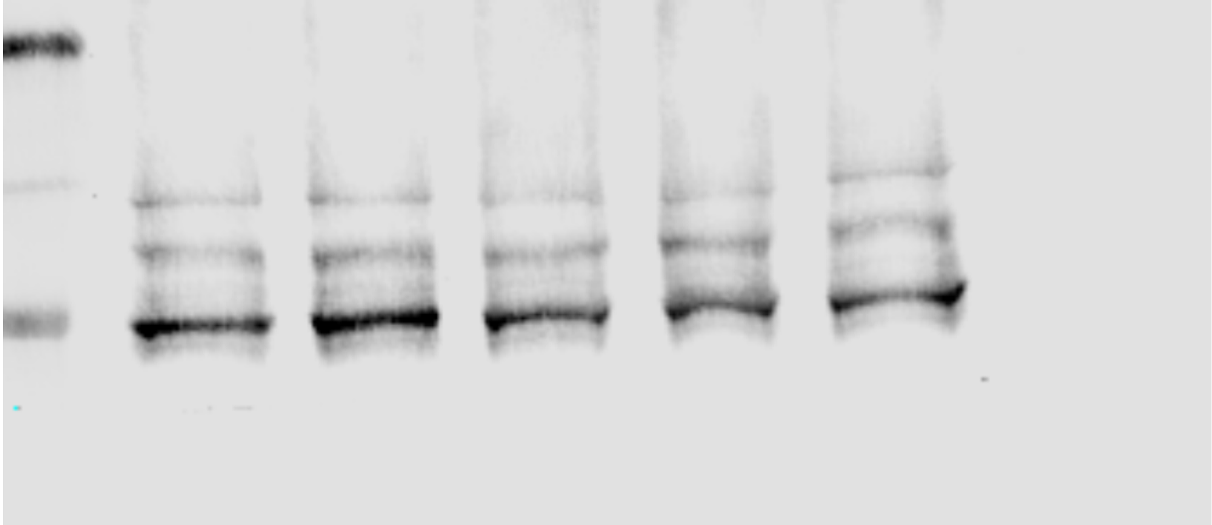

Raw Image Figure 3A: α-PGRN (AF2420)

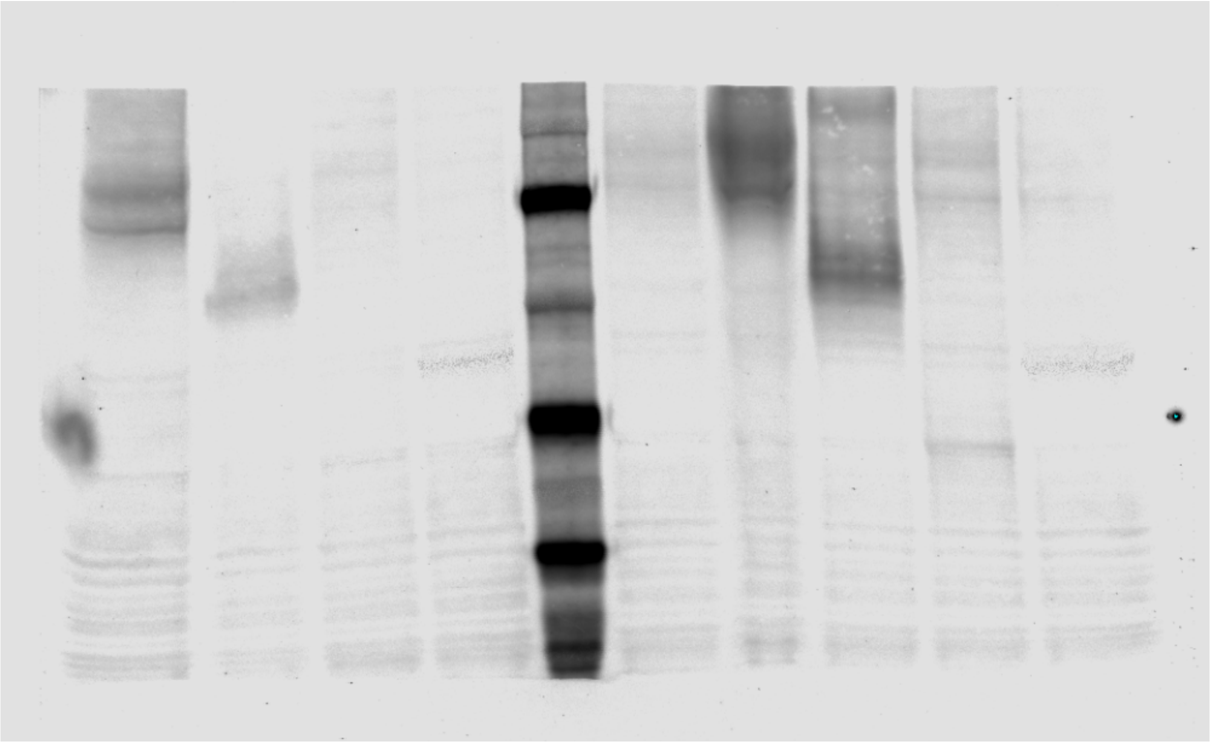

Raw Image Figure 3A: α-Flag

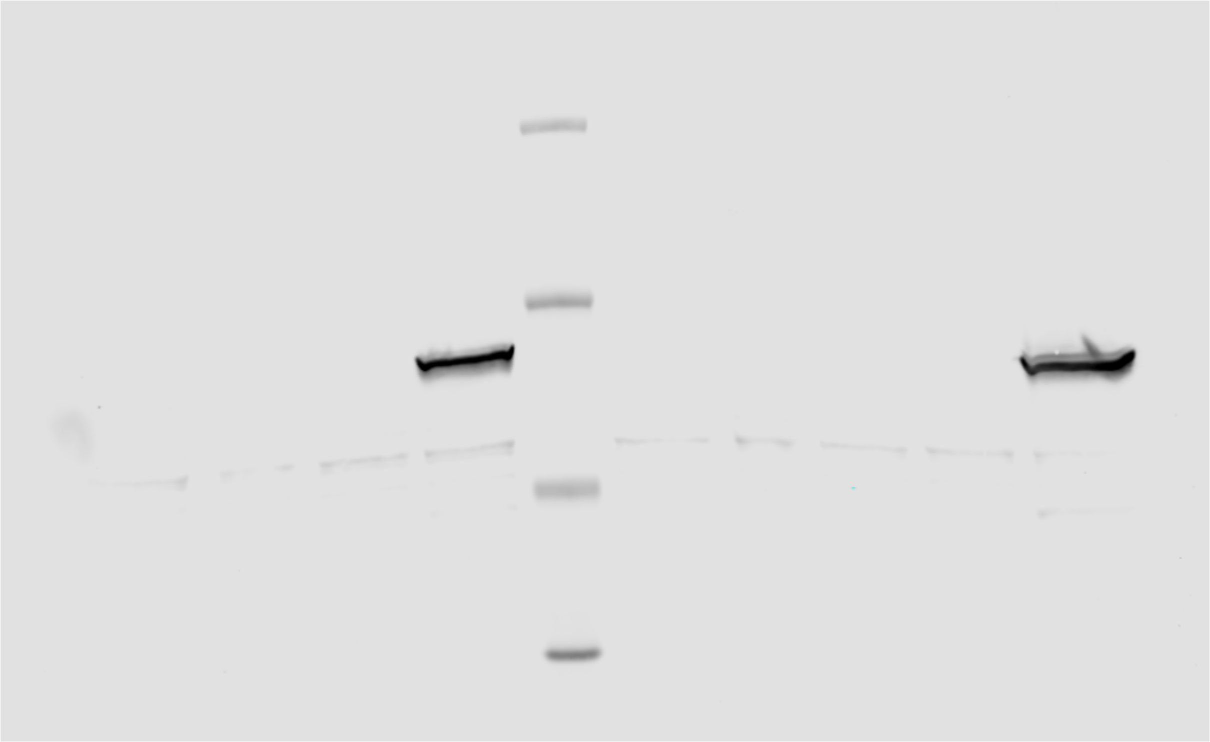

Raw Image Figure 3A: α-Vinculin

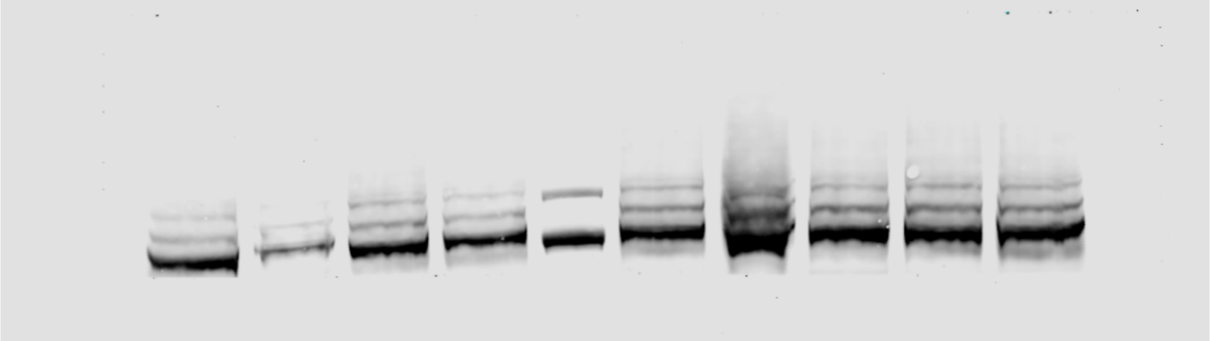

Raw Image Figure 4D: α-PGRN (AF2420)

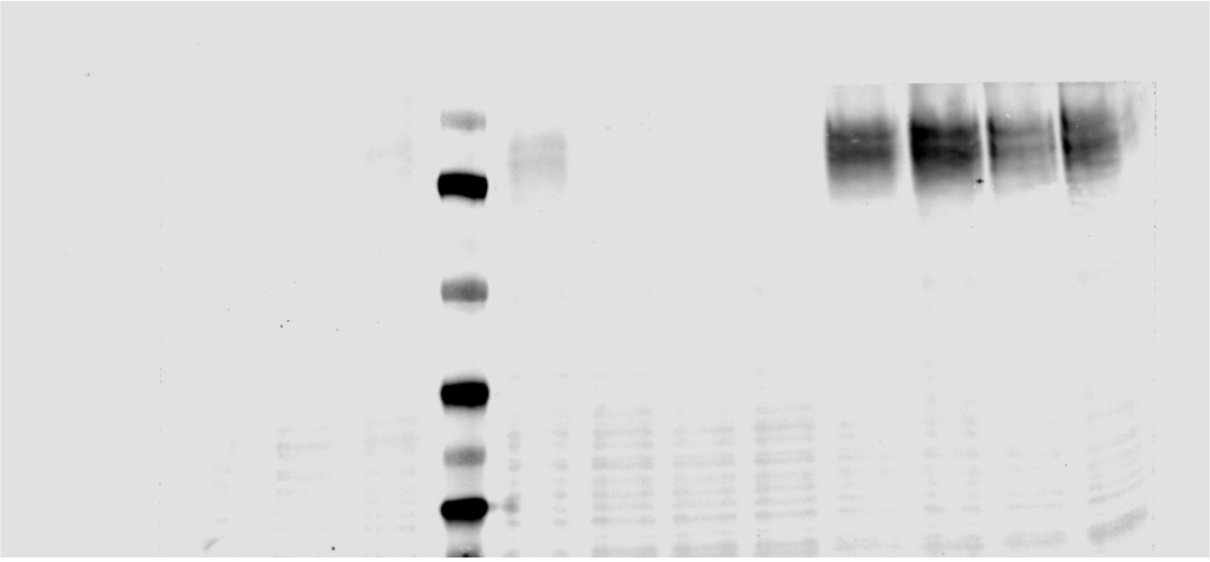

Raw Image Figure 4D: α-UPF1

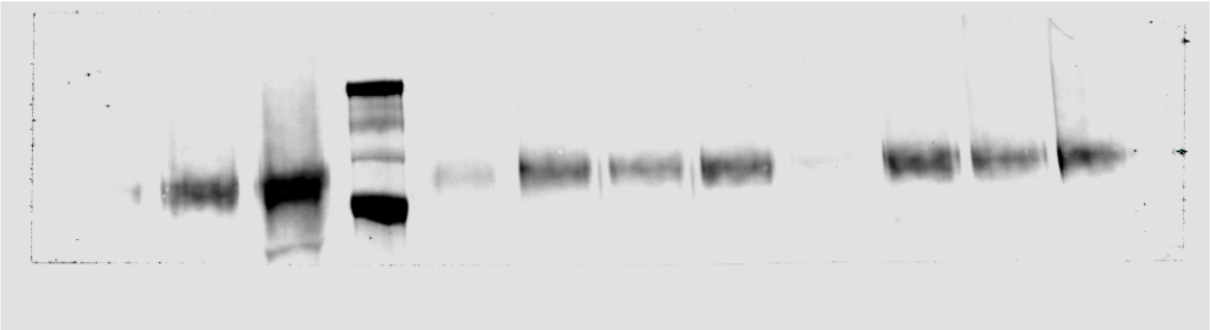

Raw Image Figure 4D: α-GAPDH

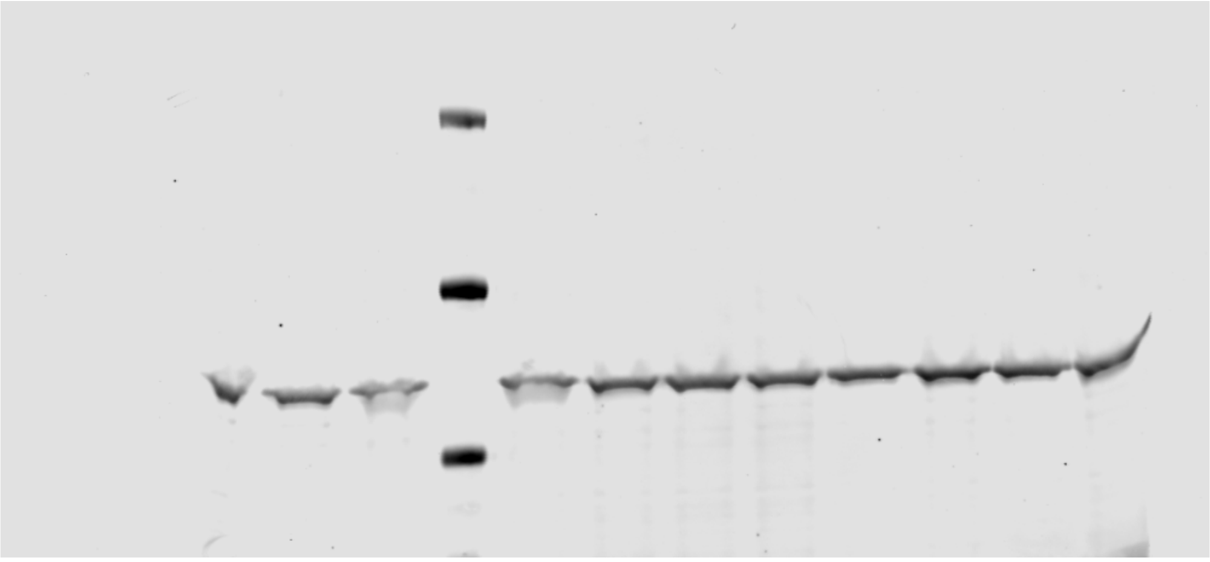

Raw Image Figure 5A: α-PGRN (AF2420, high intensity)

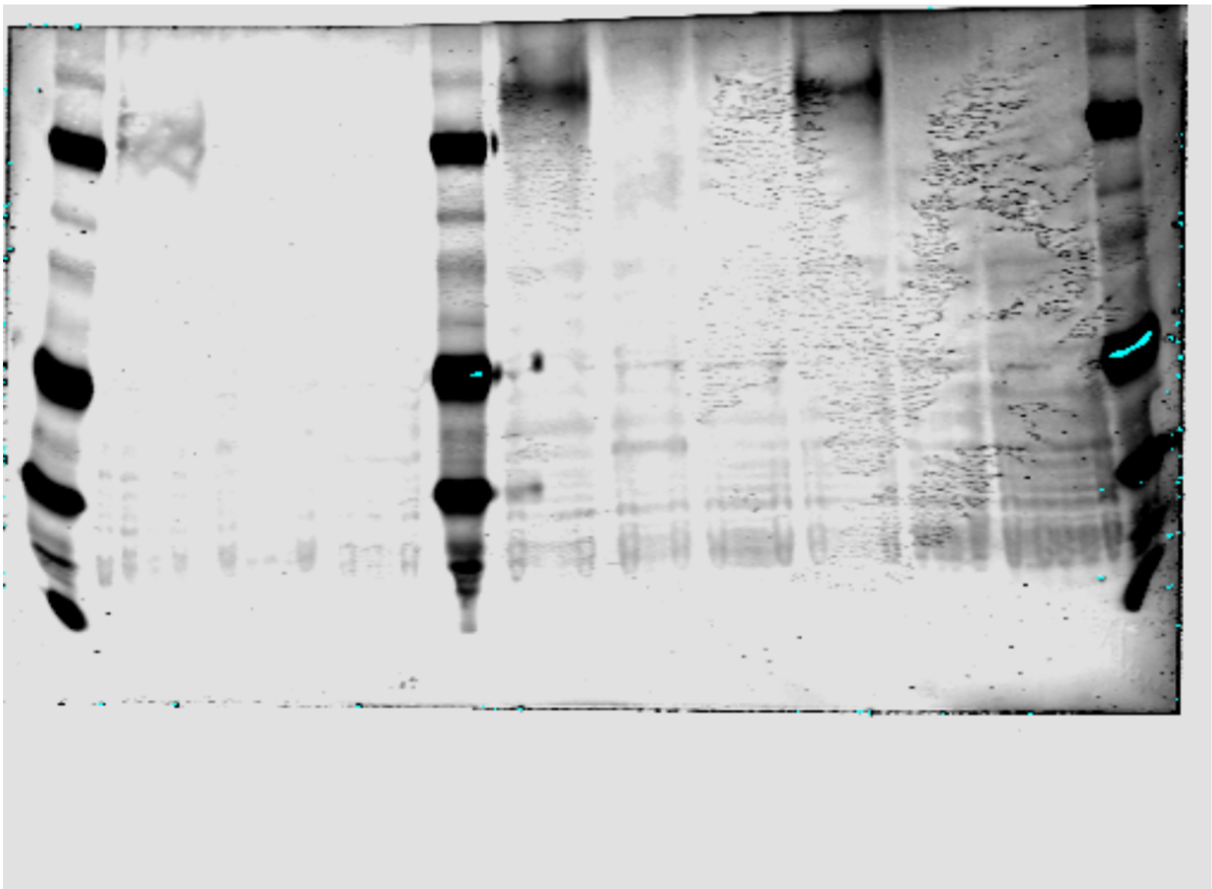

Raw Image Figure 5A: α-GAPDH

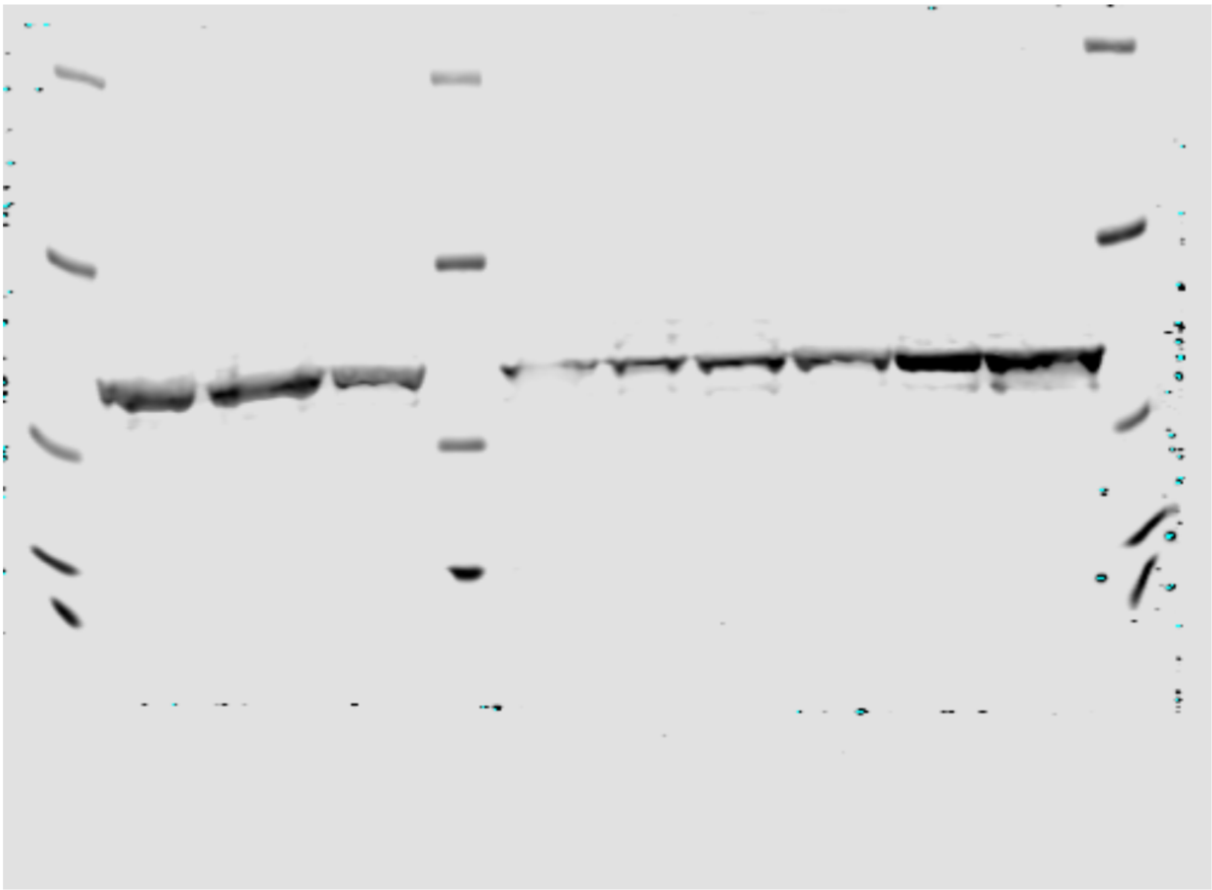

Raw Image Figure 5A: α-PGRN (AF2420, low intensity)

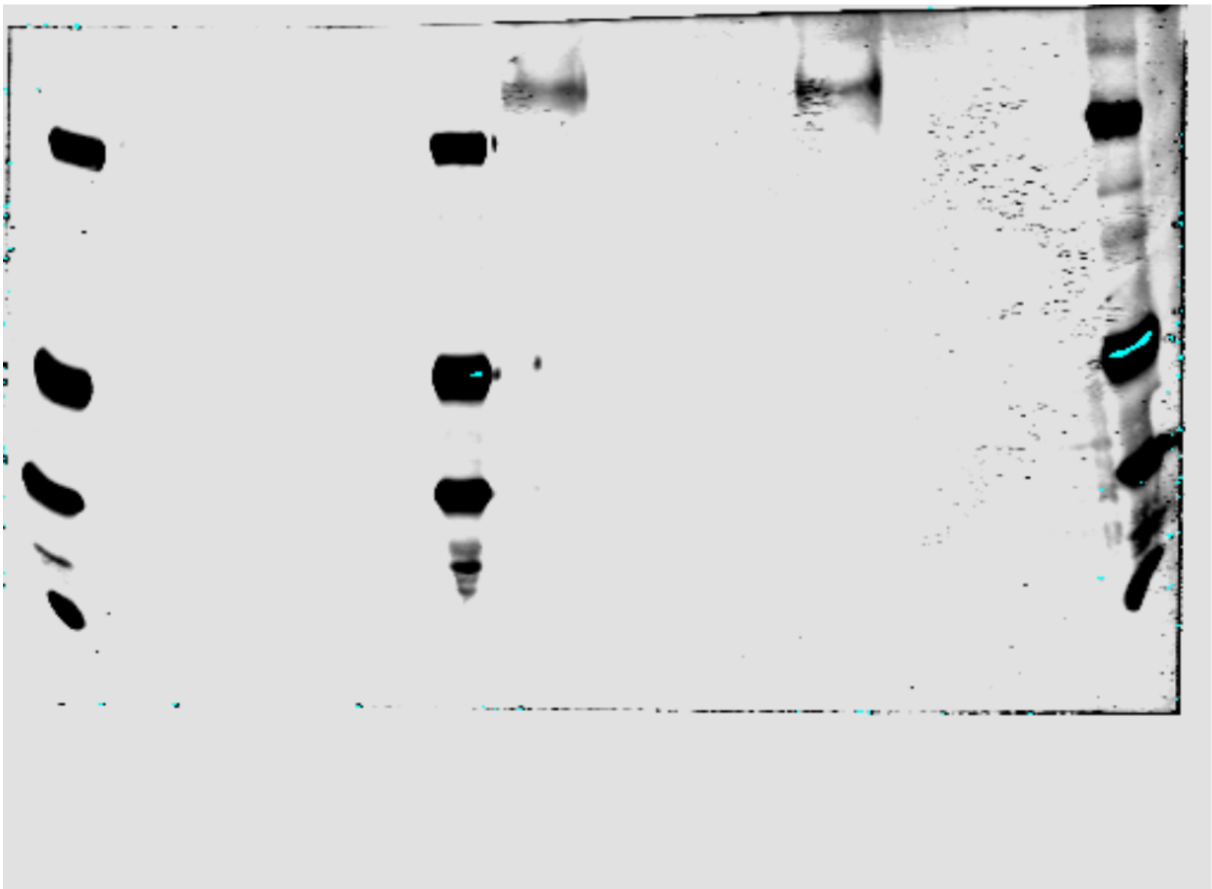

Raw Image Figure 5A: α-Vinculin

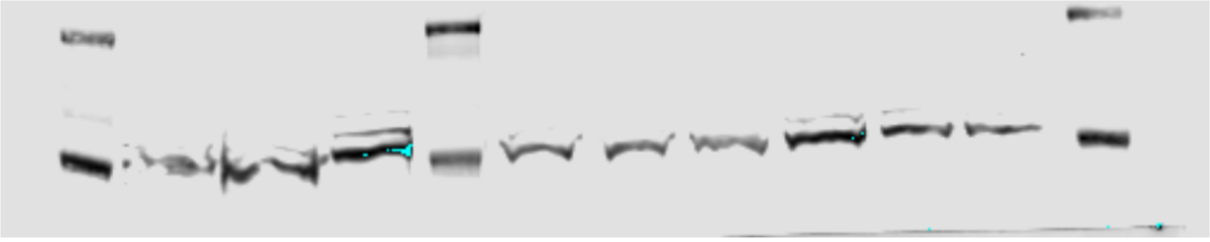

Raw Image Figure 5B: α-PGRN (AF2420)

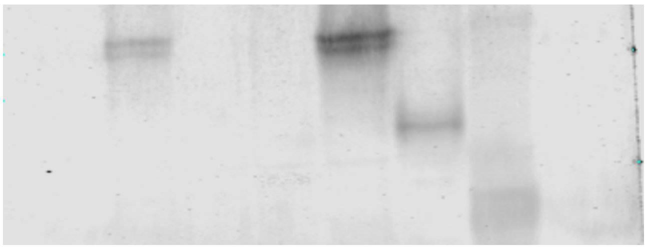

Raw Image Figure 5A: α-Flag

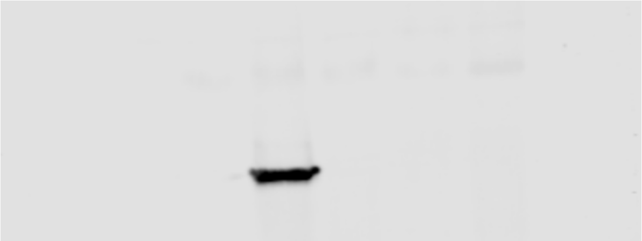

Raw Image Figure 5A: α-Vinculin

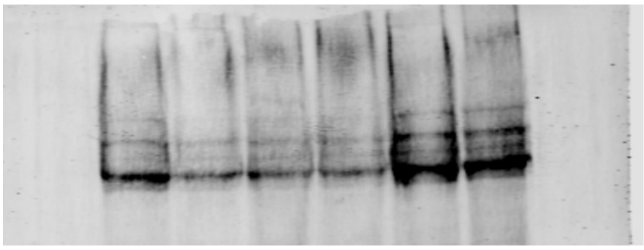

Raw Image Supplementary Figure S1B: α-PGRN (sc-377036)

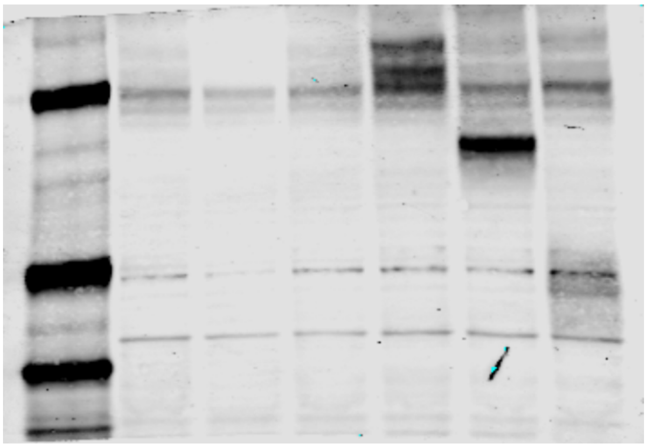

Raw Image Supplementary Figure S1B: α-Vinculin

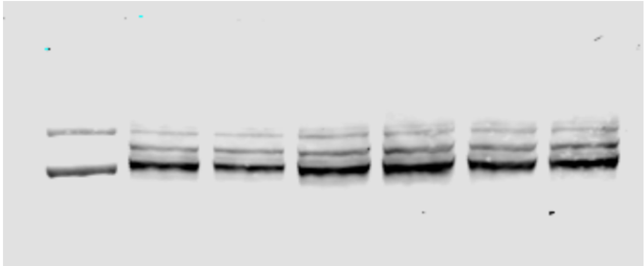

Supplement: Supplementary file 6 — Supplementary Material 6 [file 41598_2025_26703_MOESM6_ESM.pdf]
